# On the basis of sex and sleep: the influence of the estrous cycle and sex on sleep-wake behavior

**DOI:** 10.3389/fnins.2024.1426189

**Published:** 2024-08-29

**Authors:** Kevin M. Swift, Nicholas C. Gary, Phillip J. Urbanczyk

**Affiliations:** Medical Readiness Systems Biology Branch, Walter Reed Army Institute of Research, Silver Spring, MD, United States

**Keywords:** estrous cycle, sleep, sex differences, female rodents, proestrus, estrus

## Abstract

The recurrent hormonal fluctuations within reproductive cycles impact sleep-wake behavior in women and in rats and mice used in preclinical models of sleep research. Strides have been made in sleep-related clinical trials to include equal numbers of women; however, the inclusion of female rodents in neuroscience and sleep research is lacking. Female animals are commonly omitted from studies over concerns of the effect of estrus cycle hormones on measured outcomes. This review highlights the estrous cycle’s broad effects on sleep-wake behavior: from changes in sleep macroarchitecture to regionally specific alterations in neural oscillations. These changes are largely driven by cycle-dependent ovarian hormonal fluctuations occurring during proestrus and estrus that modulate neural circuits regulating sleep-wake behavior. Removal of estrous cycle influence by ovariectomy ablates characteristic sleep changes. Further, sex differences in sleep are present between gonadally intact females and males. Removal of reproductive hormones via gonadectomy in both sexes mitigates some, but not all sex differences. We examine the extent to which reproductive hormones and sex chromosomes contribute to sex differences in sleep-wake behavior. Finally, this review addresses the limitations in our understanding of the estrous cycle’s impact on sleep-wake behavior, gaps in female sleep research that are well studied in males, and the implications that ignoring the estrous cycle has on studies of sleep-related processes.

## The estrous cycle

**Figure 1:**
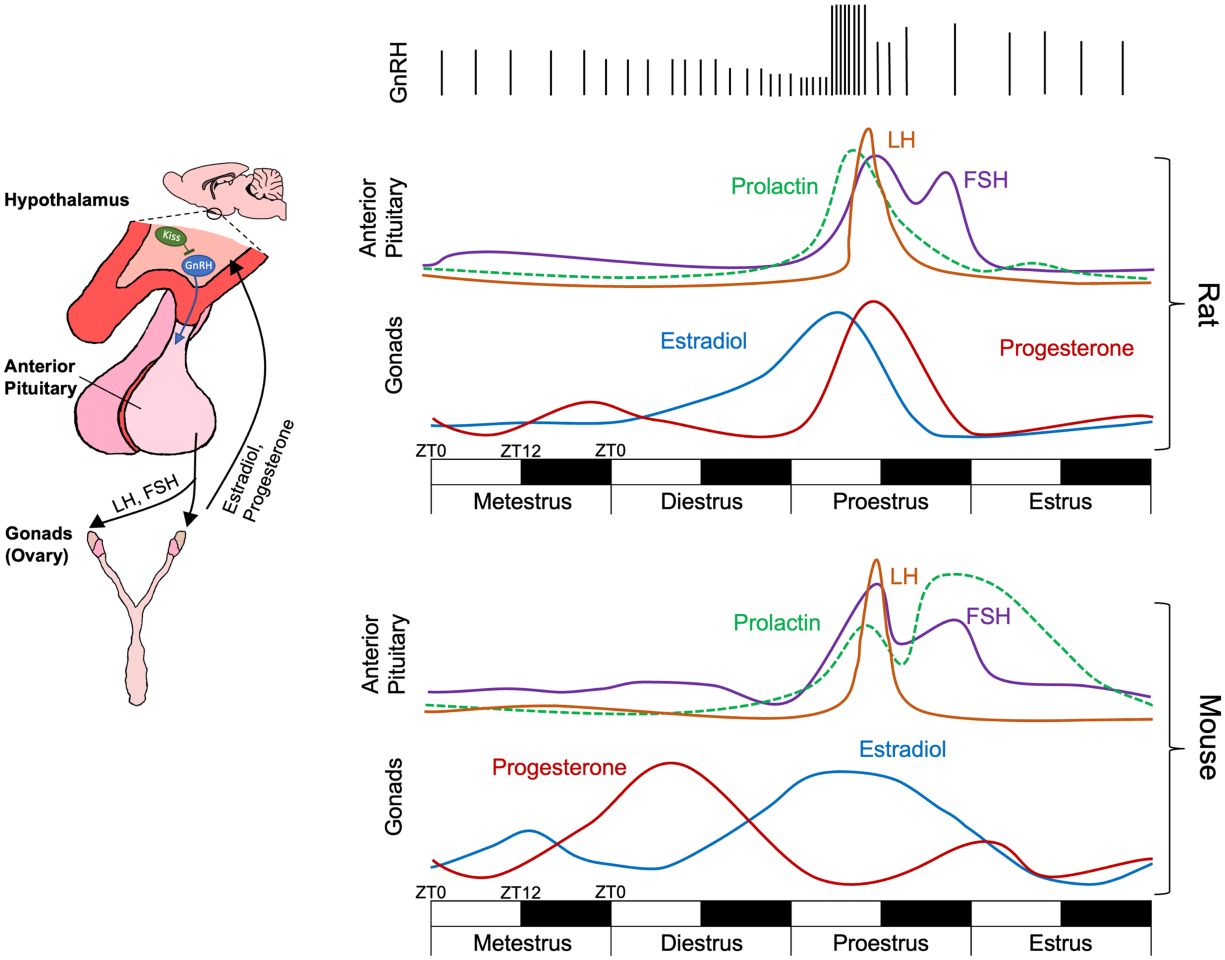
Schematic of the hypothalamic–pituitary-gonadal axis activity across the rat and the mouse estrous cycle over a four-day (96 h) period. Each day is composed of a 12-h light 12-h dark cycle with ZT0 representing lights on and ZT12 representing lights off. While cycle phases are displayed as equal in length occurring over a single 24-h (12-light, 12-dark) period, actual phase lengths vary (Paccola et al., 2013; Ajayi and Akhigbe, 2020). Further phase onset and offset is not locked to transitions between light and dark. During metestrus and diestrus, infrequent GnRH pulses cause FSH and low levels of LH release from the anterior pituitary. During proestrus, elevated estradiol increases GnRH pulse frequency, triggering a surge of LH release inducing ovulation and decreasing in estradiol. Elevated progesterone feedback to kisspeptin (Kiss) neurons which modulate GnRH pulses, decreasing their frequency. Relative hormone levels for rats taken from Scharfman and MacLusky (2006) and Smith et al. (1975) and for mice from McLean et al. (2012).

The estrous cycle is a recurrent pattern of hormonal changes that mediate fertility in several species of mammals. The recurrent cyclical progression through the phases of the estrous cycle is regulated by hormonal release from the hypothalamic–pituitary-gonadal (HPG) axis (Figure 1). Gonadotropin-releasing hormone (GnRH) neurons in the hypothalamus serve as a central common pathway for reproductive regulation in both females and males (Christian and Moenter, 2010). In females, pulsatile release of GnRH stimulates the release of luteinizing hormone (LH) and follicle stimulating hormone (FSH) from the anterior pituitary (Marshall and Griffin, 1993). LH and FSH regulate ovarian follicle maturation and the production of ovarian steroids, estradiol and progesterone. These ovarian steroids feedback on kisspeptin neurons, the upstream regulators of GnRH neurons, modulating GnRH neuron activity and controlling GnRH release (Christian and Moenter, 2010).

In female rats and mice the estrous cycle is approximately 4–5 days in length and is divided into four phases: metestrus, diestrus, proestrus, and estrus (Miller and Takahashi, 2014). During metestrus and diestrus, infrequent GnRH pulses produce FSH and low levels of LH. FSH increases ovarian follicle maturation resulting in increased release of estradiol from ovarian follicles. During proestrus, elevated estradiol increases GnRH pulse frequency, triggering a surge of LH release (Park and Ramirez, 1989; Sarkar et al., 1976). Notably, increases in GnRH pulse frequency occur during the later portion of the light phase of proestrus with the LH surge taking place within the hours adjacent to the transition to the dark phase (Chappell, 2005; Miller et al., 2004) (Figure 1). In the ovary, this LH surge induces ovulation, the release of the oocyte from the primary follicle, 10–12 h post-LH surge (Paccola et al., 2013) and is marked by a rapid decrease in estradiol. The remnants of the primary ovarian follicle become the corpus luteum, which releases progesterone and prepares the uterus for implantation should fertilization occur (Niswender, 2002; Niswender et al., 1994). Peak fertility occurs directly following ovulation and during estrus. If fertilization and pregnancy does not occur during estrus, the corpus luteum breaks down, progesterone levels decrease, and the cycle begins anew. One key difference between mice and rats is the release pattern of progesterone across the estrous cycle. While rats display an increase in progesterone following the LH surge, progesterone peaks earlier in mice during diestrus (Wood et al., 2007; McLean et al., 2012) (Figure 1).

While estrous cycle phases are commonly diagramed as being equal in length, phase length varies within and across species. Rat estrous cycle phase lengths are approximately: metestrus 6–21 h, diestrus 48–72 h, proestrus 12–14 h, and estrus 12–48 h (Paccola et al., 2013; Ajayi and Akhigbe, 2020). Meanwhile, mice estrous cycle lengths are approximately: metestrus 2–24 h, diestrus 48–72 h, proestrus less than 24 h, and estrus 12–48 h (Ajayi and Akhigbe, 2020). Common methods for determining estrous cycle in mice and rats are outlined in Byers et al. (2012) and Ajayi and Akhigbe (2020).

## The estrous cycle alters sleep-wake behavior

**Figure 2:**
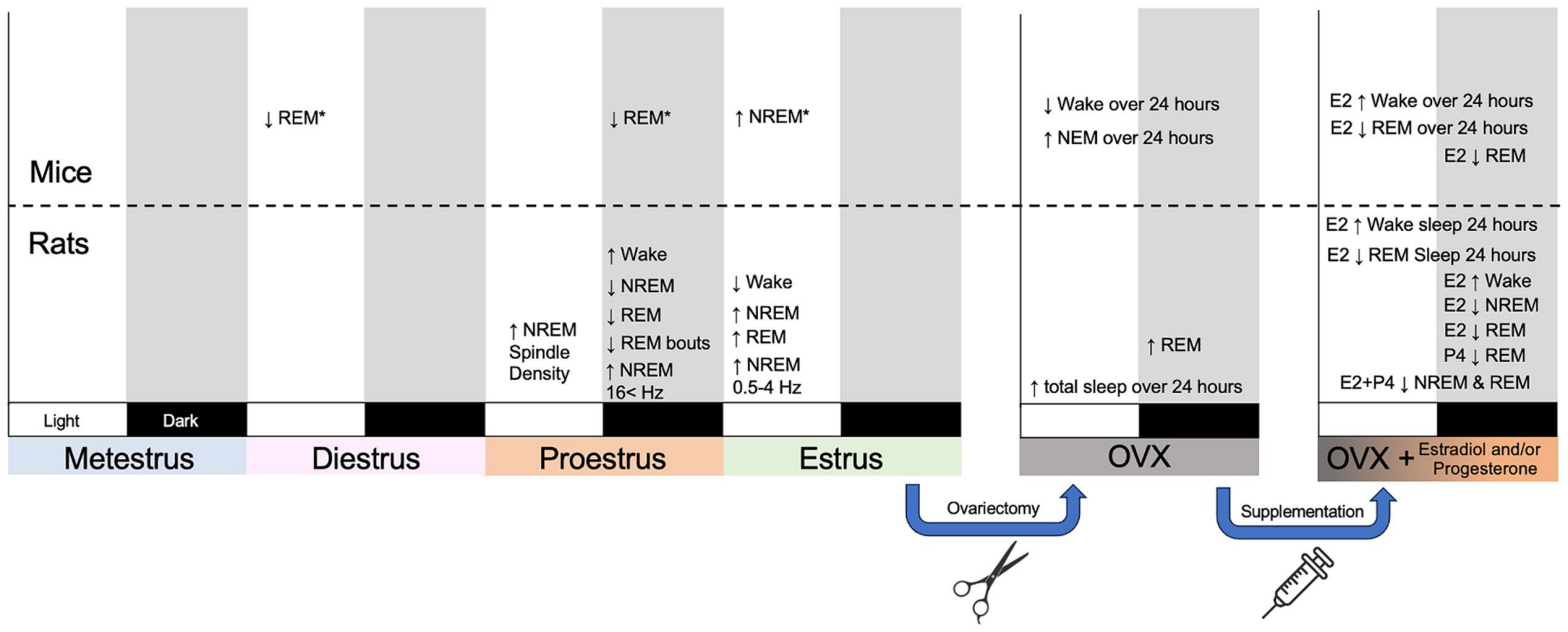
Summary figure outlining broad changes in sleep-wake behavior, sleep architecture, and sleep neural oscillations across the estrous cycle in mice and rats. The estrous cycle is displayed across a four-day period with each day encapsulating a different phase. Subsequent panels represent the effect of ovariectomy (OVX) and OVX with hormone supplementation over the course of day. Each phase has a light and a dark phase. Changes displayed in light or dark designate when they took place. Changes measured across a 24-h period rather than solely a light or a dark phase are labeled as such. E2 designated estradiol; P4 designates progesterone. *Strain specific effects in mice (Koehl et al., 2003).

Electroencephalographic recordings in the 1960’s by Sawyer and colleagues identified changes in sleep-wake behavior across the estrous cycle in female Sprague–Dawley rats (Colvin et al., 1968; Yokoyama et al., 1965). During the dark/active phase of proestrus, female rats displayed reduced amounts of non-rapid eye movement (NREM) sleep and rapid eye movement (REM) sleep. Decreased sleep was accompanied by prolonged periods of wakefulness with enhanced arousal behavior and locomotor activity compared to analogous periods in other estrous phases. These sleep-wake changes coincided with estrous phases where increases in LH and FSH occurred—suggesting that reproductive hormones modulate sleep-wake rhythms (Colvin et al., 1968). Over 50 years later, the suppression of NREM sleep and REM sleep during proestrus has been replicated in several rat strains (Fang and Fishbein, 1996; Schwierin et al., 1998; Zhang et al., 1995; Hadjimarkou et al., 2008; Swift et al., 2019; Tóth et al., 2024; Kostin et al., 2020). During the dark phase of proestrus, the length and frequency of REM sleep bouts is decreased, and REM sleep is disproportionately suppressed compared to NREM sleep. Following proestrus, some studies have identified a rebound in either NREM sleep, REM sleep, or both during the subsequent light phase of estrus (Yokoyama et al., 1965; Schwierin et al., 1998; Swift et al., 2019). This rebound sleep is more consolidated with longer bouts of NREM and REM sleep and decreased NREM bout frequency (Swift et al., 2019). Sleep changes have also been reported during metestrus and diestrus, but broadly, proestrus and estrus have the largest impact on sleep in rats (Figure 2).

The impact of the estrous cycle on sleep in mice is less defined. Early research established that sleep in mice is largely influenced by genetic background (Valatax et al., 1972). Similarly, a mouse’s genetic background determines the extent to which sleep is affected by the estrous cycle. A seminal study by Koehl et al. (2003) compared sleep in Balb/cJ, C57BL/6J, and C3H/HeJ mice across the estrous cycle. Similar to rats, C57BL/6 J mice had decreased REM sleep during the dark phase of proestrus compared to diestrus but did not show a sleep rebound during estrus. C3H/HeJ mice displayed a rebound in NREM sleep during the light phase of estrus, but also had decreased REM sleep during the light phase of diestrus. Sleep-wake behavior in Balb/cJ mice was widely unaffected by the estrous cycle. A study of orexin knock-out C57BL/6 J used to study cataplexy found that the estrous cycle had no impact on sleep in female mice (Arthaud et al., 2022). Altogether, the estrous cycle has mixed effects on sleep in mice and these effects are highly dependent on strain (Figure 2).

## Ovarian hormones produce cycle-specific changes in sleep

Early work hypothesized that ovarian release of estradiol and progesterone is necessary for the phase-dependent sleep changes during proestrus and estrus. Removing hormonal influence via ovariectomy (OVX) largely ablates the characteristic NREM and REM sleep changes seen within intact cycling females (Kleinlogel, 1983; Choi et al., 2021). However, OVX produces mixed effects on sleep-waking behavior. An initial study found that OVX rats had less REM sleep than intact female rats during estrus and diestrus (Yokoyama et al., 1965). In contrast, subsequent studies found that OVX rats had increased REM sleep during the dark phase compared to intact females (Fang and Fishbein, 1996; Kleinlogel, 1983; Li and Satinoff, 1996). One study found that OVX increased the total sleep time over a 24-h period—specifically by increasing dark phase NREM and REM sleep while decreasing waking (Colvin et al., 1969). A separate study found that OVX rats had increased NREM sleep in the light phase and less NREM in the dark phase alongside increased REM sleep during both light and dark phases (Li and Satinoff, 1996). Studies of OVX mice display decrease waking and increased NREM sleep across 24 h (Choi et al., 2021; Paul et al., 2006).

Reintroducing estradiol and progesterone into OVX animals produce sleep changes similar to those seen in proestrus and estrus (Figure 2). Further, the changes in sleep resulting from the reintroduction of ovarian hormones are more consistent than the changes following OVX. Administering estradiol to OVX rats and mice reduces REM sleep, NREM sleep, and increases waking (Choi et al., 2021; Colvin et al., 1969; Matsushima and Takeichi, 1990; Cusmano et al., 2014; Deurveilher et al., 2009, 2011; Paul et al., 2009; Schwartz and Mong, 2013; Schwartz and Mong, 2011; Pawlyk et al., 2008a,b). Moreover, these REM sleep and waking changes were most notable during the dark phase (Schwartz and Mong, 2013; Schwartz and Mong, 2011; Pawlyk et al., 2008b; Matsushima and Takeichi, 1990). Reintroducing progesterone also suppresses REM sleep during the dark phase (Deurveilher et al., 2009). The administration of estradiol and progesterone together produces an equal or synergistically greater suppression of REM and NREM sleep than estradiol alone (Deurveilher et al., 2009; Branchey et al., 1971). Taken together this suggests that elevated levels of estradiol and progesterone during proestrus are largely responsible for the suppression of sleep that normally occurs during this time.

Reproductive hormones also contribute to the development of sleep-wake circuitry in both males and females. Administering estradiol and progesterone to adult male rats that were castrated as neonates suppresses dark phase NREM and REM sleep similar to females (Branchey et al., 1973). This sleep suppression by administering ovarian hormones does not occur in males castrated as adults suggesting that the development of sleep-wake circuitry is influenced by perinatal reproductive hormones. Another study exposed neonatal females to masculinizing levels of testosterone and examined their sleep as adults (Cusmano et al., 2014). Masculinized adult females displayed reduced expression of neural activity marker c-fos in sleep-active neurons in ventral lateral preoptic area (VLPO) compared to control females (Cusmano et al., 2014). Administering ovarian hormones to these masculinized females also did not suppress sleep. Moreover, administering estradiol to females that were ovariectomized just prior to puberty or post-puberty as adults yields similar effects on sleep—suggesting that the effect of estradiol on sleep circuitry organization is specific to early development rather than puberty (Cusmano et al., 2014). As such, reproductive hormones help orchestrate the organization of sleep-wake circuitry during early development and are necessary for sleep’s modulation by reproductive hormones during puberty and adulthood.

## Mechanisms of sleep modulation by ovarian hormones

Current evidence points to several different mechanisms by which ovarian hormones regulate sleep-waking behavior. First, elevated levels of estradiol promote wakefulness by suppressing activity in sleep-promoting brain regions. Work by Mong and colleagues has demonstrated that estradiol decreases activity in sleep-active neural population in the VLPO and median preoptic nucleus (MnPN) (Mong et al., 2011; Mong and Cusmano, 2016). Administration of estradiol in OVX rats decreases c-fos expression in VLPO neurons (Hadjimarkou et al., 2008; Deurveilher et al., 2008). Additionally, estradiol suppresses sleep-promoting prostaglandin D2. This suppression is driven by estradiol decreasing both prostaglandin D2 mRNA and the enzyme (lipcaline-type prostaglandin D synthase) responsible for its synthesis in VLPO neurons (Hadjimarkou et al., 2008; Mong et al., 2003; Urade and Hayaishi, 2011; Ueno et al., 1982; Mong et al., 2003). The suppression of REM sleep specifically by estradiol is partially due to its action in the MnPN. Blocking estrogen receptors in the MnPN prevents the estradiol-induced suppression of REM sleep (Mong and Cusmano, 2016; Cusmano et al., 2011). Estradiol also promotes wakefulness by enhancing activity in arousal centers. Administering estradiol increases c-fos expression in the tuberomammillary nucleus (TMN) arousal center (Hadjimarkou et al., 2008). As the VLPO and the TMN have reciprocal inhibitory connections (Saper et al., 2011), an increase in TMN neuron activity inhibits sleep-promoting VLPO neurons. Thus, estradiol promotes arousal by inhibiting sleep-promoting neurons and activating neurons in wake-promoting regions (Hadjimarkou et al., 2008).

The mechanisms by which progesterone modulate sleep are less clear. Progesterone has sleep inducing effects in rats and humans (Lancel et al., 1997). These effects are caused by progesterone’s neuroactive metabolites, allopregnanolone and pregnenolone, acting as positive allosteric modulators of the GABA_A_ receptor, rather than progesterone acting on its endogenous receptor (Lancel et al., 1996; Lancel, 1999). This action is similar to that of benzodiazepines and is hypothesized to be the mechanism by which progesterone promotes sleep. Administering progesterone, allopregnanolone, or pregnanolone in male rats decreases NREM sleep latency (Lancel et al., 1996, 1997; Edgar et al., 1997). In contrast, administering progesterone to female rats, particularly with or following daily doses of estradiol, promotes wakefulness and suppresses sleep (Deurveilher et al., 2009; Branchey et al., 1971). The impact of progesterone’s influence on sleep when administered with estradiol is confounding, but perhaps necessary. Progesterone receptor expression is dependent on estrogen receptor gene expression (Quadros and Wagner, 2008); i.e., the absence of estradiol decreases progesterone receptor expression. This complicates studies that reintroduce solely progesterone in OVX animals and see no effect on sleep as progesterone receptor density is likely diminished without estradiol. Another consideration is the release pattern of progesterone between mice and rats. In rats, estradiol and progesterone release is largely concurrent during proestrus, whereas mice display maximal progesterone release earlier during diestrus (McLean et al., 2012; Scharfman and MacLusky, 2006). This separate release of estradiol and progesterone in mice allows a more granular evaluation of the impact of ovarian hormones and sleep-wake activity. However, it may also contribute to more variation on how sleep changes across the estrous cycle in mice.

A separate hypothesis suggests that progesterone suppresses sleep by increasing body temperature (Boivin and Shechter, 2010). Progesterone decreases neural activity in preoptic heat-sensitive neurons and increases activity in preoptic cold-sensing neurons, resulting in a net upward shift in the thermoregulatory set point and an overall increase in body temperature (Nakayama et al., 1975). Elevated levels of endogenous progesterone during the estrous cycle and the menstrual cycle increase body temperature (Marrone et al., 1976; Driver et al., 1996). Administration of exogenous progesterone elicits similar effects (Marrone et al., 1976; Driver et al., 1996; Stachenfeld et al., 2000). Increases in body temperature are associated with sleep disruption, REM sleep suppression, and insomnia (Lack et al., 2008). Women report the highest incidence of sleep disturbances during the luteal phase preceding menstruation when progesterone levels peak and core body temperature is elevated (Manber and Bootzin, 1997). However, the effect of progesterone on human sleep is also mixed. Administration of progesterone decreases sleep disturbances in postmenopausal women and increases NREM sleep in men (Caufriez et al., 2011; Friess et al., 1997; Schüssler et al., 2008; Wiedemann et al., 2017). However, progesterone receptor agonist megestrol acetate decreases REM sleep in men in an inverted U-shaped manner, i.e., high and low doses suppress REM to a lesser magnitude than medium doses (Wiedemann et al., 1998). Future work is necessary to better understand the mechanisms by which progesterone alters sleep.

Finally, ovarian hormones may also influence sleep by altering neuromodulatory output from arousal centers. The cholinergic basal forebrain, dopaminergic ventral tegmental area, noradrenergic locus coeruleus, serotonergic dorsal raphe, and the orexinergic lateral hypothalamus all modulate sleep-wake behavior. Moreover, optogenetic activation of these brain regions promotes wakefulness and prevents sleep (Carter et al., 2010; Zant et al., 2016; Eban-Rothschild et al., 2016; Moriya et al., 2021; De Luca et al., 2022).

Progesterone and estradiol receptors are expressed within these neuromodulator centers (Mong and Cusmano, 2016; Shughrue et al., 1997; Curran-Rauhut and Petersen, 2002) and their receptor density can fluctuate across the estrous cycle (Haywood et al., 1999). These fluctuations in receptor density, and ovarian hormones, likely impact neuromodulator tone. Furthermore, these neuromodulator centers also regulate mating behavior and sexual receptivity (Uphouse, 2014; Melis and Argiolas, 1995; Clemens et al., 1989; Muschamp et al., 2007; Etgen and Morales, 2002). Although the interplay of neuromodulators is more complex in mating behavior, maximal sexual receptivity in females occurs following ovulation during the night of proestrus where longer bouts of wake are common (Powers, 1970). It is likely that elevated neuromodulatory tone promotes both the increased wakefulness and sexual receptivity—potentially to maximize mating opportunities. However, there is a paucity of studies recording electrical activity or calcium transients from these arousal centers throughout the estrous cycle and further studies are necessary to determine how they are influenced by ovarian hormones.

## The effect of prolactin on sleep

Prolactin is a peptide hormone released from the anterior pituitary which is responsible for regulating lactation in mammals (Bachelot and Binart, 2007). Prolactin release changes across the estrous cycle with levels remaining low through metestrus and diestrus, but increase dramatically during proestrus and remain elevated through estrus, particularly in mice (McLean et al., 2012; Freeman et al., 2000). In mice, prolactin release displays a brief pre-ovulatory increase before reaching its highest levels during estrus (McLean et al., 2012). In rats, peak prolactin release coincides with the LH surge during proestrus. Rats prolactin levels also display a diurnal rhythm with increased release during sleep (Sassin et al., 1972; Bethea and Neili, 1979). Moreover, the increased release of prolactin during sleep is dependent on sleep state rather than the time of day (Sassin et al., 1973). Prolactin levels are highest during NREM sleep, and decrease during REM sleep (Parker et al., 1974) due to modulation by hypothalamic dopamine (Freeman et al., 2000). Low dopamine during NREM sleep allows prolactin release while increased dopamine during REM sleep inhibits release (Spiegel et al., 1994). Despite prolactin’s release pattern during sleep and across the estrous cycle, little is known about the interplay between prolactin, sleep, and the estrous cycle phase. In rats, prolactin levels are higher during the dark phase than the light phase (Dunn et al., 1976; Laakso et al., 1988; Roky et al., 1995; Wong et al., 1983). Injections of prolactin during the light phase increase the amount of REM sleep, whereas administration during the dark phase increases waking and suppresses REM sleep—specifically by decreasing the number of REM sleep bouts (Roky et al., 1993, 1994). This REM sleep suppression is notable as it suggests that elevated prolactin may contribute to the decrease in REM sleep during dark phase of proestrus. However, to our knowledge, there is no study examining the effect of exogenous prolactin on sleep in either in gonadally-intact female rats or mice across the estrous cycle or in OVX animals.

## Estrous cycle & sleep neural oscillations

Delta waves or slow waves are high amplitude 0.5–4 Hz oscillatory activity which occur within NREM sleep as the result of the simultaneous activation and subsequent inactivation of large neural populations. Slow waves are functionally important for sleep-dependent memory consolidation, cerebrospinal fluid circulation, and metabolite clearance in the brain during sleep (Xie et al., 2013; Huber et al., 2004; Fattinger et al., 2017; Boespflug and Iliff, 2018). Although studies in women have found no difference in NREM slow wave power or slow wave activity (SWA) across the menstrual cycle (Driver et al., 1996; Baker and Lee, 2018; Driver et al., 2008), NREM SWA does change across the estrous cycle in rats. A study of female rats found SWA was decreased during proestrus compared to other estrous phases (Schwierin et al., 1998), with SWA activity reaching the lowest level during the transitions period from the light to the dark phase. In contrast, another study found that SWA activity was increased in multiple brain regions during the dark phase of proestrus compared to all other estrous phases (Swift et al., 2019). However, both studies identified increased NREM delta power during the light phase of estrus. In mice there was no effect of estrous cycle phase or mouse strain (Balb/cJ, C57BL/6 J, C3H/HeJ) on NREM SWA (Koehl et al., 2003).

Hippocampal ripples are transient (~100 msec) periods of 100–250 Hz high frequency oscillatory activity that result from the activation of hippocampal pyramidal neurons primarily during NREM sleep and waking inactivity (Wilson and Mcnaughton, 1994; Roux et al., 2017; Jadhav et al., 2012). During sleep, ripples are important for the replay and stabilization of hippocampal memories encoded during waking (Girardeau et al., 2009; Gridchyn et al., 2020). Our understanding of hippocampal ripples in humans comes from studies of seizure patients implanted with focal electrodes (Wixted et al., 2014, 2018; Staresina et al., 2015); however, none of these studies have compared differences in ripple density or frequency between men and women. Numerous studies have recorded ripples from rodent hippocampi, but to our knowledge, no study exists comparing ripple characteristics in rats or mice between sexes or within females across the estrous cycle. One factor that could impact ripples is adenosine; activation of adenosine receptors decreases ripple incidence (Ortiz and Gutiérrez, 2015; Jarosch et al., 2015). Female rats display increased CA1 adenosine concentrations during proestrus compared to other cycle phases and males (Borgus et al., 2020), which could cause cycle-dependent variations in ripple incidence. Additionally, there is substantial evidence that ovarian hormones alter memory function and hippocampal cell morphology. Changes in estradiol and progesterone levels across the estrous cycle alter CA1 synaptic function and spine density (Woolley and McEwen, 1993; Woods and McEwen, 1992) and between sexes, estradiol acts differentially to potentiate synapses in males versus females (Oberlander and Woolley, 2017; Jain et al., 2019). Hippocampal memory function also changes across the estrous cycle with elevated levels of estradiol associated with enhanced memory performance (Luine, 2014). As ovarian hormones have broad and differing effects on hippocampal processes, future work should consider ripples and memory replay when examining sex differences in hippocampal function.

Sleep spindles are transient (0.5–2 s) of 10–15 Hz neural oscillatory activity that wax and wane in amplitude. Sleep spindles are generated during NREM sleep by reciprocal inter- and intra-circuit connections between thalamic and cortical neural circuits (Steriade et al., 1993; Clawson et al., 2016). In humans, rodents, and other mammals, spindles have been implicated in the consolidation of memories (Iotchev et al., 2017; Swift et al., 2018; Fogel and Smith, 2006; Mednick et al., 2013); either directly or via the coupling of spindles with NREM slow waves and hippocampal ripples (Siapas and Wilson, 1998; Wierzynski et al., 2009; Maingret et al., 2016). Women have a higher sleep spindle density (spindles/min) than men, with a greater percentage of spindles occurring in the left frontal cortex (Gaillard and Blois, 1981; Huupponen et al., 2002). Additionally, spindle density, length, frequency of spindle oscillations, and their spectral power change across the menstrual cycle (Driver et al., 1996; Ishizuka et al., 1994; De Zambotti et al., 2015). In female rats, spindle density increases during the light phase of proestrus compared to diestrus and estrus and the frequency of spindle oscillations peak during the light phase of proestrus and metestrus (Swift et al., 2019). Changes in spindles across the estrous cycle may be partially attributed to fluctuations in progesterone. Administration of progesterone and allopregnanolone in rats increases NREM sleep spindle power and the amount of intermediate or pre-REM sleep—a spindle-rich state that often precedes REM sleep in rats (Lancel et al., 1996, 1997). However, future work is necessary to confirm the potential impact of reproductive hormones on sleep spindles.

The beta (16–30 Hz) and gamma (30–100 Hz) bands are less commonly studied during sleep as they are often associated with waking or insomnia (Perlis et al., 2001). Menstrual cycle phase has a mild effect on NREM sleep beta with higher beta power during the luteal phase compared to the follicular phase (Driver et al., 1996). This study did not examine frequencies greater than 25 Hz, so it is unclear whether the menstrual cycle alters sleeping gamma power. However, NREM beta and gamma power are affected by the estrous cycle (Schwierin et al., 1998; Swift et al., 2019) with increases in both frequency bands during the dark phase of proestrus in rats. In mice, REM sleep gamma (30–120 Hz) is increased during estrus compared to diestrus (Barth et al., 2014). The increase in higher frequency bands (>16 Hz) during sleep may be partially attributed to elevated levels of progesterone. Administration of progesterone and allopregnanolone in rats increase 16–40 Hz spectral power during NREM and REM sleep (Lancel et al., 1996, 1997). Further, progesterone increases NREM beta power in men (Friess et al., 1997). The functional significance of increases in the beta and gamma bands during the dark phase of proestrus remains unknown but may represented increased waking pressure, potentially to maximize reproductive opportunities following ovulation.

REM sleep theta (5–9 Hz) emanates from the hippocampus and is generated by cholinergic and parvalbumin interneuron projections from the medial septum to the hippocampus (Ognjanovski et al., 2017; Simon et al., 2006). Frequency power within the REM sleep theta band varies across the estrous cycle phase (Schwierin et al., 1998). However, changes in REM theta were of small magnitude, regionally-specific, and occurred at isolated times within the cycle (Swift et al., 2019). The impact of OVX and hormone replacement on REM sleep theta is mixed. One study found that neither ovariectomy nor administering estradiol, progesterone, or both to OVX rats affected REM sleep theta power (Deurveilher et al., 2011). In contrast, four days of estradiol treatment reduced REM sleep theta power in OVX rats during the dark phase (Pawlyk et al., 2008a). Another study found that administering progesterone to male rats reduced 7–8 Hz theta in a dose dependent manner (Lancel et al., 1996). Together these studies demonstrate that while ovarian hormones may affect REM sleep theta further work is necessary to understand their relationship.

## Sleep homeostasis and the estrous cycle

The homeostatic rebound in sleep amount and SWA following extended periods of wake is well documented in rodents and humans (Choi et al., 2021; Borbely and Achermann, 1999). SWA rebound following sleep deprivation is increased in women compared to men (Armitage and Hoffmann, 2001; Hajali et al., 2019). To our knowledge, no study has examined SWA rebound following sleep deprivation during specific menstrual phases; although different phases of the menstrual cycle are more prone to sleep disturbances (Manber and Bootzin, 1997). In mice, females show a greater rebound in NREM sleep following sleep deprivation compared to males (Paul et al., 2006). However, gonadectomy (GDX) attenuates the increased NREM sleep rebound when comparing GDX males and GDX females. Four days of REM sleep deprivation produces REM rebound in male and in female rats regardless of estrous phase. However, females entering proestrus following REM deprivation were the slowest to recover as demonstrated by prolonged period of REM sleep rebound (Andersen et al., 2008). It is likely that rats undergoing REM recovery during proestrus had prolonged recovery due to competing mechanisms: REM rebound following deprivation and REM suppression commonly seen during proestrus. In contrast, the SWA response to sleep deprivation is largely unaffected by the estrous cycle. Sleep deprivation during the light phase of proestrus or estrus produces a similar SWA response (Schwierin et al., 1998). However, ovarian hormones themselves can alter the response to sleep deprivation. Treating OVX females with estradiol, progesterone or a combination attenuates the SWA response to sleep deprivation (Deurveilher et al., 2009). All treatment groups displayed normal NREM and REM sleep rebound, but OVX rats treated with solely estradiol had a greater relative increase in REM sleep recovery.

## Circadian influences on the estrous cycle

Light–dark (LD) cycles serve as cues which entrain molecular biological pacemakers. While all tissues contain pacemaker activity, the suprachiasmatic nuclei (SCN) in the hypothalamus is broadly the master pacemaker (Miller and Takahashi, 2014; Mohawk et al., 2012). Within the estrous cycle, both the release of ovarian hormones and proper SCN circadian signaling are necessary to elicit GnRH surges triggering ovulation. Lesioning the SCN abolishes circadian release of GnRH and produces aovulatory cycles (Wiegand and Terasawa, 1982). Ablation of ipsilateral SCN inputs to kisspeptin expressing neurons, the upstream regulators of GnRH neuron activity, decreases c-fos expression in GnRH neurons (Smarr et al., 2012). Further, bursts of GnRH neural activity which trigger the LH surge are under circadian regulation. In gonadally-intact females, LH surges are largely confined to the end of the light phase of proestrus (Miller and Takahashi, 2014; Chappell, 2005). Treating OVX female rats chronically with estradiol produces LH surges on multiple days, but notably, the surges are restricted to the end of the light phase (Legan and Karsch, 1975). Even altering the LD cycle does not greatly change the time within a day when LH surges occur. Animals housed under different LD cycles (e.g., L:D 12:12, 16:8) display LH surges at similar times in relation to the increased activity occurring at dark phase onset—regardless of the length of the LD cycle (Christian and Moenter, 2010; Moline et al., 1981). Moreover, ovulation occurs at similar times within the day regardless of whether animals are kept on a LD cycle, constant light, or constant dark (Mccormack and Sridaran, 1977).

Circadian rhythms also play a role in fertility and pregnancy. Circadian disruption can detrimentally affect reproductive health, particularly in women where it can cause irregular menstrual cycle and decreased fertility (Lawson et al., 2011; Wang et al., 2016; Lawson et al., 2015; Mínguez-Alarcón et al., 2017). Circadian disruption also affects the estrous cycle in rats and mice (Miller and Takahashi, 2014). Rotating LD cycles every 3, 6, or 12 days to model shift work increases the estrous cycle length in mice (Yoshinaka et al., 2017). Further, the magnitude of cycle disruption and the proportion of mice with disrupted cycles is dependent on the frequency of LD reversals. Mutations to the *Clock* gene, a key component of the molecular pacemaker, can also be used as a model of circadian disruption. *Clock* mutant females display dampened LH surges (<1.8 ng compared to >10 ng in controls), and loss of cyclicity of the estrous cycle compared to controls (Miller et al., 2004). When pregnant, *Clock* mutants have a higher rate of fetal resorption and failure to deliver the pups at full term (Miller et al., 2004). These pregnancy complications occur in the presence of no change in breeding behavior between mutants and controls. A similar relationship between circadian disruption and pregnancy complications in women has also been reported. A study of over 20,000 women found that night work the week prior increases the risk of miscarriage in a dose dependent manner (Begtrup et al., 2019)—suggesting that the detrimental effects of circadian disruption are well conserved across species.

## Sex differences in sleep: sex chromosomes vs. gonadal function

Sleep in gonadally-intact males and females is most notably different during proestrus and estrus when females have less NREM and REM sleep and more waking compared to males (Fang and Fishbein, 1996; Swift et al., 2019; Kostin et al., 2020). Proestrus also marks sex differences in sleep microarchitecture, particularly in REM sleep. Even sex differences studies that do not account for the estrous cycle still identify differences between male and female sleep (Choi et al., 2021; Paul et al., 2006). These sex differences are largely dependent on gonadal function and the majority of differences disappear between sexes in GDX rats and GDX mice (Paul et al., 2006; Cusmano et al., 2014). Hormone replacement via testosterone treatment to GDX males and estradiol to GDX females largely restore sex differences in sleep and waking (Choi et al., 2021; Paul et al., 2009).

Despite this, not all sex differences in sleep disappear between male and female GDX rats and mice (Paul et al., 2006; Cusmano et al., 2014). GDX male mice have increased REM sleep recovery and decreased NREM delta power following sleep deprivation compared to GDX female mice (Paul et al., 2006). Another study found that GDX males have increase waking, and decreased NREM and REM sleep during the dark phase compared to GDX females (Choi et al., 2021). Interestingly, replacing endogenous hormones in GDX rats does not have equal effects across GDX males and female rats. Administering estradiol to GDX females suppresses sleep, particularly REM sleep, whereas infusion of testosterone into GDX males has little effect on sleep (Cusmano et al., 2014). Together these results suggest that sex differences in sleep are caused by factors outside of gonadal hormones, such as sex chromosomes.

The four-core genotype model is a tool that tests the influence of sex chromosomes or sex-linked traits independent of gonadal development and function. Within the model the SRY gene [whose absence or presence determines gonadal development regardless of sex chromosome (Berta et al., 1990; Sekido and Lovell-Badge, 2009)] is added to XX-females causing the development of male gonads. Similarly, the SRY gene is removed from XY-males to produce female gonads (Ehlen et al., 2013). The result is four genotypes: XY-male (+SRY, testes, male), XY-female (-SRY, ovaries, female), XX-male (+SRY, testes, male), and XX-female (-SRY, ovaries, female). One study used the model to determine whether sex chromosomes account for sex differences in sleep which persist after GDX (Ehlen et al., 2013). The study found that gonadally intact XX-females and XY-females have less NREM sleep during the dark phase compared to XY-males and XX-males. Following gonadectomy, GDX XX-males had more sleep than GDX XX-females during the dark phase. There was no differences between GDX XY-males or GDX XY-females (Ehlen et al., 2013). Finally, following 6hours of sleep deprivation, GDX XY-females had increased amounts NREM sleep and enhanced NREM SWA in comparison to GDX XX-females, suggesting Y-linked traits are responsible for increased homeostatic response to sleep deprivation. This study provides the first evidence that both hormones and sex chromosomes contribute to overall sex differences in sleep.

The four-core genotype model is currently only available in mice although a rat model is in preliminary development (Arnold et al., 2023). One consideration for the four-core genotype study (Ehlen et al., 2013) is that it utilized C57BL/6 J mice which show limited changes in sleep across the estrous cycle, but the authors dismissed these changes and did not control for the estrous phase (Paul et al., 2006). As estrous phase has a greater impact on sleep in rats, performing the same experiments in rats using a four-core genotype approach as well as controlling for the estrous phase may provide further insight. Preliminary results from the rat four-core model suggest that XY-female and XX-female rats both display normal estrous cycles with high fertility so replication of previous work in rats may yield novel results (Arnold et al., 2023).

## Considerations and future directions

### Sleep and memory consolidation

Extensive work supports the relationship between sleep and memory formation (Rasch and Born, 2013; Poe, 2017), as well as between ovarian hormones and memory formation (Luine, 2014). However, the intersection between hormonally driven sleep-wake changes and their influence on sleep-dependent memory consolidation remain unexplored. Previous studies have shown that memory formation and learning strategies are affected by estrous phase (Korol et al., 2004; Kirry et al., 2018). Memory improvements, particularly during proestrus have been attributed to increased estradiol promoting synaptic plasticity in hippocampal circuits, but the impact of sleep is uncertain. Increased spindle density during the light phase of proestrus and a suppression of REM during the dark phase represent physiologically relevant periods where variations in synaptic plasticity may alter memory consolidation (Fang and Fishbein, 1996; Schwierin et al., 1998; Swift et al., 2019). The suppression of sleep during the dark phase of proestrus, and the subsequent sleep rebound during the morning of estrus present unique periods to examine how physiological changes in sleep may impact sleep-dependent memory processes. For instance, females in the dark phase of proestrus may perform poorly on REM sleep-dependent tasks as REM sleep is almost completely suppressed during that time. The enhanced spindle density in the mPFC during proestrus may also serve to improve memory function—potentially by improving hippocampal connectivity via enhanced spindle-ripple coupling. Future work in gonadally-intact and OVX females should examine the impact estrous phase and ovarian hormones on sleep oscillations and how they impact sleep dependent memory consolidation.

### Sleep and post-traumatic stress

Post-traumatic stress disorder (PTSD) and other anxiety disorders are more common in women compared to men (Kessler et al., 2017). Among other factors, this difference in prevalence has been partially attributed to changes in ovarian hormone levels promoting resilience or susceptibly to psychological stressors. Low hormone levels in the early follicular phase are purported to cause enhanced fear extinction in women compared to those in the late-follicular phase when hormone levels are elevated (Milad et al., 2006). In rats, females display increased levels of fear extinction retention during proestrus when estradiol and progesterone are elevated (Milad et al., 2009). Moreover, administering estradiol and/or progesterone to female rats in metestrus significantly improved fear extinction recall (Milad et al., 2009). In parallel, a separate body of work suggests that sleep disruption also contributes to mental health issues. Rats with disrupted sleep display enhanced anxiety-like behaviors and insomnia is bidirectionally related to anxiety in humans (Alvaro et al., 2013; Grubac et al., 2019; Krause et al., 2017). Despite both hormonal milieu and sleep impacting mental health, little work has examined the intersection of how sleep changes due to ovarian hormone fluctuations contribute trauma-related mental health disorders. Sleep disturbances preceding or following a trauma increase the likelihood of developing mental health disorders, particularly PTSD (Bryant et al., 2010; Koren et al., 2002). Sleep disturbances are commonly reported during the late luteal phase and menstruation in women (Baker and Driver, 2007). Further, sleep disturbances are also common during the transition to menopause when ovarian hormones decrease. These periods of disturbed sleep where ovarian hormone levels decrease may be potential windows of increased vulnerability to trauma, and as such, may contribute to the increased incidence of PTSD in women. However, few studies have evaluated this intersect. Only a single study in male rats (Vanderheyden et al., 2015) examined sleep in models of PTSD and no study has examined how estrus-related sleep changes impact behavior in PTSD models.

### Combatting a male bias

Despite mandates for the inclusion of both sexes in future studies, the discrepancy between sexes persists today—particularly in neuroscience (Shansky, 2019; McCarthy et al., 2017; Shansky and Woolley, 2016). A reoccurring motif throughout this review is the absence of citable studies using female rodents. Indeed, even within the field of sleep research several *fundamental* physiological processes (e.g., sleep neural oscillatory activity) have not been studied in female rodents, much less female rodents across the estrous cycle. These fundamental physiological processes are often assumed to be 1) equivalent to those occurring in males or 2) unaffected by the estrous cycle. This assumption does a disservice to the field by promoting a male bias, and more perniciously, in some instances may be entirely false. Further, as these fundamental physiological processes (e.g., hippocampal ripples) impact higher order processes (e.g., memory consolidation) it becomes evident that a significant majority of our understanding of the neuroscience of sleep is solely derived from the male brain.

The reluctance to include female animals in studies partly stems from concern that females and estrus cycle hormones will increase variability on a measured outcomes (Shansky, 2019). However, previous studies have shown that including females does not inherently increase variability. Rocks et al. (2022) demonstrated that when not controlling for estrous cycle phase, certain behaviors are not significantly different between males and females. In the same merit, controlling for estrous phase may identify a previously masked sex difference or further elucidate an existing sex difference that is apparent when the cycle is not controlled for. While the inclusion of female animals and systematic estrous cycle tracking presents some challenges their inclusion in study design enhances relevancy and the scientific merit of the results.

## Conclusion

The estrous cycle and reproductive hormones modulate sleep-wake behavior and play a vital role in the development of sleep neural circuitry. From sex chromosomes to gonads, reproductive processes influence female sleep creating a sex-specific milieu that is often quite distinct from males. Despite this, our understanding of sleep-wake processes is categorically biased towards males. While significant strides have been made towards including female animals in research, we have just begun to scratch the surface of studying foundational neurological processes in female animals. Researchers must view the inclusion of female animals in preclinical studies not as a burden, but as an opportunity and a necessity to those who their research informs. Doing so will holistically improve our understanding of sleep physiology alongside the more important goal of better understanding and improving women’s health.
